# Treatment-Seeking for Children with Suspected Severe Malaria Attending Community Health Workers and Primary Health Centres in Adamawa State, Nigeria

**DOI:** 10.3389/ijph.2025.1607697

**Published:** 2025-09-26

**Authors:** Tristan T. Lee, Elizabeth Omoluabi, Kazeem Ayodeji, Ocheche Yusuf, Charles Okon, Nina C. Brunner, Giulia Delvento, Aita Signorell, Mark J. Lambiris, Marek Kwiatkowski, Christian Burri, Christian Lengeler, Emmanuel Emedo, Fatima Cheshi, Chizoba Fashanu, Owens Wiwa, Manuel W. Hetzel

**Affiliations:** ^1^ Swiss Tropical and Public Health Institute, Allschwil, Switzerland; ^2^ University of Basel, Basel, Switzerland; ^3^ Akena Associates, Abuja, Nigeria; ^4^ UNICEF, Abuja, Nigeria; ^5^ Clinton Health Access Initiative, Abuja, Nigeria

**Keywords:** case fatality, primary health care, severe febrile illness, malaria, quality of care

## Abstract

**Objectives:**

In the context of introducing pre-referral rectal artesunate for children with severe malaria in Adamawa State, Nigeria, case fatality was 19% among severely sick children visiting primary health centres (PHCs), and 6% among those visiting community health workers (CORPs). To understand this difference, we investigated illness severity, treatment-seeking, and background characteristics of these patients.

**Methods:**

589 children <5 years of age with fever and danger signs indicative of severe malaria attending CORPs (N = 314) and PHCs (N = 275) were enrolled. After 28 days, caregivers were interviewed about background characteristics, symptom severity, and treatment seeking practices; children attending CORPs and PHCs were compared.

**Results:**

Compared to children attending CORPs, those attending PHCs were more likely to live in urban areas, have ≥4 clinical danger signs (50% vs. 39%, p = 0.02) and signs of central nervous system involvement (90% vs. 74%, p < 0.01), and more often received initial home treatment (42% vs. 33%, p = 0.04).

**Conclusion:**

PHCs may see sicker children due to community assumptions of their professional capacity. Health system strengthening is required to capacitate PHCs to handle severe conditions and save lives in rural communities.

## Introduction

Nigeria accounts for 26% of the world’s malaria cases and 31% of malaria deaths [1]. The country is challenged with high levels of poverty, a large rural population with few transportation options, and a generally poorly functioning health system, particularly at the rural primary care level [2].

Uncomplicated malaria may progress to severe disease and death if it is not appropriately treated [3–5]. This process can occur quickly; therefore, early diagnosis and prompt, effective management are essential [6]. Initial malaria treatment is generally provided by primary health centres (PHC) and, in several countries in sub-Saharan Africa and beyond, by community health workers [7]. In Nigeria, community health workers, known as Community Oriented Resource Persons (CORPs), implement the integrated community case management (iCCM) guidelines and primary health centers (PHCs) implement the integrated management of childhood illnesses (IMCI) algorithm. Both, CORPs and PHCs serve as first point-of-care; for severe conditions, both providers are supposed to refer children directly to a hospital. According to guidelines, both of these providers treat children with uncomplicated malaria with oral artemisinin-based combination therapy (ACT), while children with suspected severe malaria are referred to health facilities with inpatient departments for comprehensive clinical management [6]. Referral facilities are few and often more difficult to access, particularly from remote rural areas.

In an observational study, the Community Access to Rectal Artesunate for Malaria (CARAMAL) project investigated the introduction of rectal artesunate (RAS) as pre-referral treatment for children with suspected severe malaria in Nigeria, the Democratic Republic of the Congo, and Uganda [8]. RAS products were recently pre-qualified by WHO [9], several years after a clinical trial found pre-referral RAS to reduce death or permanent disability when administered to children under the age of 6 years with suspected severe malaria who delayed referral to a higher level health facility [10]. In Nigeria, the CARAMAL study did not find a positive health effect of introducing pre-referral RAS in three Local Government Areas (LGA) of Adamawa State. The study also found that the case fatality rate (CFR) among children reporting with signs of severe malaria was higher in children attending a PHC (18.5%) compared to those attending a CORP (5.7%) [9].

Mortality from malaria is affected by several factors, including the *Plasmodium* species, transmission intensity and vector control measures, age and health status of a person, and the effectiveness of antimalarial treatment [11, 12]. In the context of introducing pre-referral RAS in Nigeria, this study focused on the latter two aspects. The severity of a child’s condition, approximated by the presence of clinical danger signs or caregiver’s perceptions, may determine and explain treatment seeking [13]. Conversely, prompt treatment seeking is also critical to prevent progression to severe disease and death [12]. In Nigeria, among children who sought professional care for fever, only 68% did so within 24 h of illness onset [14] and home treatments for malaria are reported to be poor [15]. Costs and distance to a health facility are barriers to pursuing appropriate treatment [16, 17].

While a prompt visit to a community-based provider, such as a CORP or PHC, can accelerate treatment initiation for common conditions, severely ill patients may need to be referred to a higher level provider for appropriate care. In the CARAMAL project, two critical aspects of a functioning continuum of care for severe malaria cases, namely referral completion and post-referral treatment, were often not implemented according to guidelines, as described elsewhere [18, 19]. However, neither of these system shortfalls seem to provide an explanation for the difference in CFR between the two groups of patients [9].

Understanding reasons for the differences in CFR between children who visited CORPs and those who visited PHCs as community level providers is crucial to strengthen primary healthcare and reduce child deaths. We investigate patients enrolled in the CARAMAL project, focusing on the type and severity of symptoms, home treatment and treatment seeking delay, while taking into account background characteristics of the patients.

## Methods

### Study Design

This investigation was part of the CARAMAL project, an observational study that accompanied the roll-out of pre-referral RAS into existing iCCM and IMCI systems, as detailed elsewhere [8]. This analysis includes a cohort of children with suspected severe malaria who were prospectively enrolled by community-based providers (CORPs or PHCs) in Nigeria and followed-up at their household 28 days after enrolment.

### Setting

Study sites were Fufore, Mayo-Belwa and Song Local Government Areas (LGAs) of Adamawa State in northeastern Nigeria. The estimated total population of these LGAs was 746,950 in 2018, of which 130,430 (17%) were under the age of 5 years [20]. The population in the three LGAs is served by over 500 CORPs, 77 PHCs, and 3 referral health facilities (Cottage Hospitals). In the study area, malaria occurs seasonally.

CORPs and PHCs provide community-level primary healthcare services. The PHCs are usually small facilities with nurses, midwives and lower cadre staff. In contrast, CORPs are commonly not health professionals but lay persons in the community with a minimal training in basic healthcare provision. CORPs obtain medical supplies and supervision from nearby PHCs or NGOs. CORPs were introduced by the local health authorities in 2014 to fill an accessibility gap and serve hard to reach communities more than 5 km away from a health facility.

### Data Collection

Children under 5 years identified as cases of “severe malaria” at a PHC, or visiting a CORP with a history of fever and at least one general danger sign indicative of suspected severe malaria according to Nigerian iCCM guidelines (unusually sleepy or unconscious, not able to drink or feed, vomiting everything, convulsions, or yellow eyes), were enrolled between June 2018 and July 2020. A malaria rapid diagnostic test (mRDT) was conducted for study purposes. CARAMAL staff attempted to reach both types of providers weekly by phone to identify and register children meeting the inclusion criteria. Follow-up visits were done 28 days later in-person at the child’s residence, or by phone during the COVID-19 pandemic lockdown. Caregiver interviews were conducted in the local language (Hausa or Fulfulde) about the child’s health status, signs and symptoms of disease, treatment seeking perceptions and practices, and medicines the child received. Geo-locations of the child’s home were recorded. Children who died were followed up 2 to 3 months after enrolment to respect the mourning period. Additional details about the circumstances of the death were recorded. Data was collected electronically on password protected tablets using ODK Collect, and saved on a secure ODK Aggregate server hosted at the Swiss Tropical and Public Health Institute in Switzerland.

### Data Analysis

Study participants were included in the analysis if they fulfilled all inclusion criteria including at least one iCCM danger sign and a positive mRDT at enrolment, a successful follow-up on day 28, and written consent provided by their caregiver. Children who died more than 31 days after enrolment were excluded from the analysis.

This study compares children attending a CORP with children attending a PHC. The main variables of interest were home treatment, treatment seeking delay, presence of danger signs and reasons for attending the CORP or the PHC. Home treatment was coded as a categorical variable. Treatment seeking delay was defined as the reported number of days between illness onset and attending the CORP or PHC, categorized into two-day periods. We used the presence of danger signs involving the central nervous system (convulsions, unusually sleepy or unconscious), number of danger signs (both general iCCM danger signs used as part of the inclusion criteria and other Nigeria-specific danger signs), and caregiver-perceived severity as proxies for disease severity. Danger signs were consolidated from health worker reports at enrolment and caregiver information at day 28. Binary variables were derived to account for key time periods that may have influenced outcomes: the RAS roll-out period (starting in May 2019), the COVID-19 movement restriction period (1st April 2020 to the end of study in July 2020), and the rainy season (May to October). Living in a rural or urban setting was determined based on the child’s residence geo-location and the LandScan HD: Nigeria version 1.1 geodatabase (Oak Ridge National Laboratory, 2018) settlement patterns defined by Weber et al. [21].

Descriptive analyses investigated differences between patients attending a CORP versus those attending a PHC. Logistic regression models with clustered standard errors (clustering at the level of the enrolling healthcare provider) were adjusted for the COVID-19 pandemic lockdown period that was likely to affect treatment seeking. Clustered standard errors were used because cluster membership determines outcome [22]. P-values were calculated using the likelihood ratio test for categorical variables and on the odds ratio for binary variables. P-values of <0.05 were considered statistically significant. Unadjusted models are presented as supplementary materials. Statistical analyses were conducted in Stata SE version 16.1.

## Results

### Study Population

CORPs and PHCs provisionally enrolled 724 children. Of those, 66 (9%) were lost to follow-up or the caregiver did not provide consent and 33 (5%) did not fulfill the inclusion criteria (7 had no history of fever, 26 no danger sign at enrolment). Two children who died >31 days after enrolment were excluded, as well as 34 who had no positive mRDT at enrolment (Supplementary Figure S1).

The final analysis included 589 children with suspected severe malaria, of which 314 (53%) attended a CORP and 275 (47%) a PHC (Table 1). They were enrolled at 139 different providers (108 CORPs, 31 PHCs). CORPs enrolled fewer patients per provider (median: 1, IQR: 1-2, range: 1–38) compared to PHCs (median: 5, IQR: 1–12, range: 1–42).

**TABLE 1:** Patient characteristics by type of provider visited (Adamawa State, Nigeria. 2018–2020).

	CORP		PHC		AOR^a^	95% CI^a^	p-value^a^
	N	%	N	%			
Total	314		275
Child sex							
Male	188	60	164	60	Ref.		
Female	126	40	111	40	1.0	(0.7–1.3)	0.91
Child age (years)							
0	40	13	27	10	Ref.		0.34
1	86	27	74	27	1.3	(0.7–2.6)	
2	82	26	82	30	1.5	(0.8–2.9)	
3	68	22	55	20	1.3	(0.6–2.4)	
4	38	12	37	13	1.5	(0.7–3.4)	
Caregiver sex							
Male	102	32	95	35	Ref.		
Female	212	68	180	65	0.9	(0.6–1.3)	0.48
Caregiver age							
<25	42	13	46	17	Ref.		0.17
25–34	167	53	128	47	0.7	(0.4–1.2)	
35–45	75	24	72	26	0.8	(0.5–1.5)	
>45	30	10	28	10	0.9	(0.4–1.7)	
Missing	0	0	1	0	-		
Caregiver education^b^							
Never attended school	54	17	65	24	Ref.		0.77
Primary or lower	15	5	26	9	1.5	(0.6–3.4)	
Secondary or higher	28	9	44	16	1.3	(0.6–2.6)	
Quranic	22	7	39	14	1.5	(0.7–3.1)	
Missing	195	62	101	37	-		
LGA							
Fufore	143	46	94	34	Ref.		0.02
Mayo-Belwa	95	30	154	56	2.3	(0.5–10.4)	
Song	76	24	27	10	0.5	(0.1–1.9)	
Residence							
Rural	298	95	227	83	Ref.		
Urban	14	4	43	16	4.6	(1.4–15.4)	0.01
Missing	2	1	5	2	-		
Day of enrolment							
Workday	241	77	239	87	Ref.		
Weekend	73	23	36	13	0.5	(0.3–0.9)	0.02
Season							
Dry season	83	26	66	24	Ref.		
Rainy season	231	74	209	76	1.0	(0.6–1.6)	0.95
RAS implementation phase							
Pre-RAS	156	50	61	22	Ref.		
Post-RAS	158	50	214	78	3.3	(1.7–6.4)	<0.01
COVID-19 pandemic							
Pre-COVID-19	268	85	207	75	Ref.		
COVID-19 period	46	15	68	25	1.9	(1.0–3.6)	0.04

^a^ Logistic regression adjusting for the COVID-19 pandemic period, with standard errors clustered at the level of the health care provider. Likelihood ratio test used to calculate p-values for categorical variables.

^b^ Data not collected during the entire study period.

### Patient Characteristics

Overall, there were more male (n = 352) than female (n = 237) patients in the study and the mean patient age was 2.0 years. Caregivers were mostly female and between 25 and 34 years old. More children were enrolled in the rainy season (n = 440) than the dry season (n = 149). There were no differences in the aforementioned aspects between patients enrolled by CORPs and PHCs (Table 1, unadjusted estimates Supplementary Table S1). Children attending PHCs were more likely to live in an urban area (adjusted odds ratio [AOR]: 4.6, 95% CI: 1.4–15.4) and less likely to visit the facility during a weekend (AOR: 0.5, 95% CI: 0.3–0.9). They were also more often enrolled after the introduction of RAS in the study area than before (AOR: 3.3, 95% CI: 1.7–6.4), and during the COVID-19 pandemic (AOR: 1.9, 95% CI: 1.0–3.6) that occurred after the introduction of RAS.

### Signs and Symptoms

**FIGURE 1:**
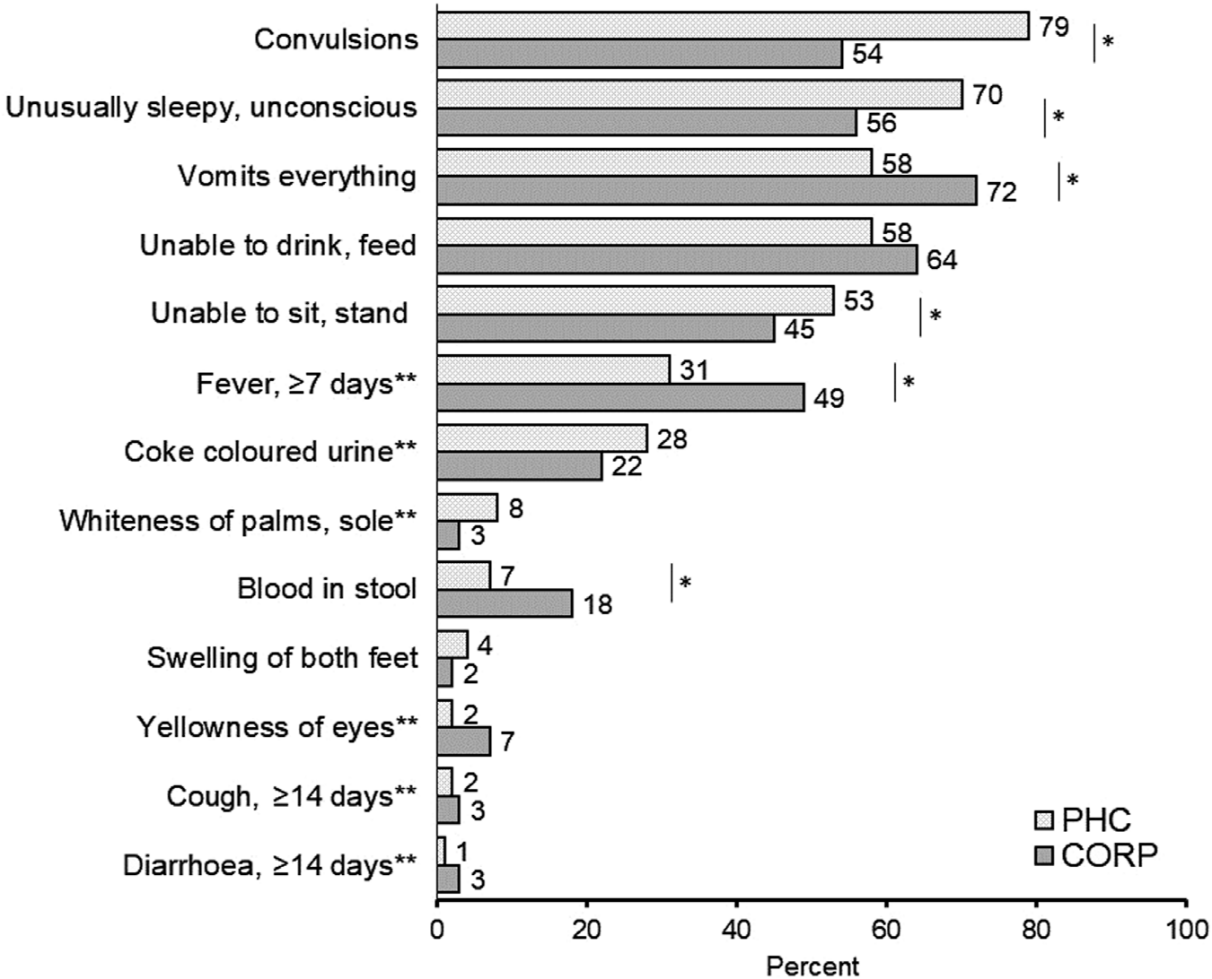
Signs and symptoms of severity during the child’s case of severe illness stratified by community-based health provider visited (Adamawa State, Nigeria. 2018–2020). *p < 0.05. **Data not collected during the entire study period.

Convulsions (79%) and being unusually sleepy or unconscious (70%) were more common in children visiting a PHC (Figure 1). Together, these symptoms involving the Central Nervous System (CNS) were reported more frequently in children visiting a PHC (90%) than in children visiting a CORP (74%) (AOR: 3.5, 95% CI: 1.9–6.1). There was no significant difference in the proportion of children with CNS symptoms during the COVID-19 pandemic (79%) compared to prior to the pandemic (82%).

Children enrolled at CORPs were more often reported to vomit everything (72%) or be unable to drink or feed anything (64%). Vomiting everything, prolonged fever (≥7 days) and blood in the stool were reported significantly more frequently among children attending a CORP (Figure 1).

Children often presented with multiple symptoms. More children attending a PHC (50%) had ≥4 danger signs compared to children attending a CORP (39%) (p = 0.02) (Table 2, unadjusted estimates Supplementary Table S2). Three-quarters of caregivers perceived their child’s illness to be serious but not fatal, with no statistically significant difference between the two compared groups.

**TABLE 2:** Number of danger signs and caregiver’s perceived severity of the illness (Adamawa State, Nigeria. 2018–2020).

	CORP		PHC		AOR^a^	95% CI^a^	p-value^a^
	N	%	N	%			
Total	314		275				
Number of danger signs^c^							
0–1^b^	52	17	32	12	Ref.		0.02
2–3	138	44	106	39	1.4	(0.8–2.4)	
≥4	124	39	137	50	2.0	(1.2–3.4)	
Perceived severity of the illness							
Not fatal	236	75	193	70	Ref.		
Fatal	76	24	79	29	1.3	(0.8–2.0)	0.25
Don’t know/missing	2	1	3	1	-		

^a^ Logistic regression adjusting for the COVID-19 pandemic, with standard errors clustered at the level of the health care provider. Likelihood ratio test used to calculate p-values for categorical variables.

^b^ 3 children were not said to have any danger signs by caregivers at follow-up but health providers noted danger signs at enrolment.

^c^ Only includes danger signs collected across the whole study period.

### Home Treatment

Treatment provided at home prior to attending a healthcare provider was more common among patients attending a PHC than those attending a CORP (CORP: 33%, PHC: 42%) (Table 3, unadjusted estimates Supplementary Table S3). Treatments included modern medicines, traditional medicines or herbs, and washing or sponging to cool the fever. Caregivers who were able to name the medicine provided prior to attending a health provider most often mentioned paracetamol (CORP: 85%, PHC: 92%) and occasionally the first-line antimalarial artemether-lumefantrine (CORP: 15%, PHC: 14%). The type of home treatment did not differ between the two groups.

**TABLE 3:** Actions taken at home prior to consulting health providers (Adamawa State, Nigeria. 2018–2020).

	CORP		PHC		AOR^a^	95% CI^a^	p-value^a^
	N	%	N	%			
Total	314		275				
Any home treatment							
No	209	67	157	57	Ref.		
Yes	103	33	116	42	1.5	(1.0–2.1)	0.04
Missing	2	1	2	1	-		
Actions taken	**103** ^b^		**116** ^b^				
Traditional medicines/herbs	22	21	26	22	1.1	(0.5–2.2)	0.86
Tepid sponging	7	7	17	15	2.4	(0.9–6.4)	0.09
Modern medicine	79	77	85	73	0.8	(0.5–1.5)	0.55
Medicines given (if known)	**67** ^b^		**76** ^b^				
Artemether- lumefantrine	10	15	11	14	1.0	(0.3–3.2)	0.99
Oral rehydration solution (ORS)	1	1	1	1	0.8	(0.1–13.4)	0.89
Paracetamol	57	85	70	92	2.1	(0.6–7.0)	0.23
Other	6	9	5	7	0.7	(0.2–2.5)	0.56

^a^ Logistic regression adjusting for the COVID-19 pandemic, with standard errors clustered at the level of the health care provider.

^b^ Denominator for this variable.

### Treatment Seeking

**FIGURE 2:**
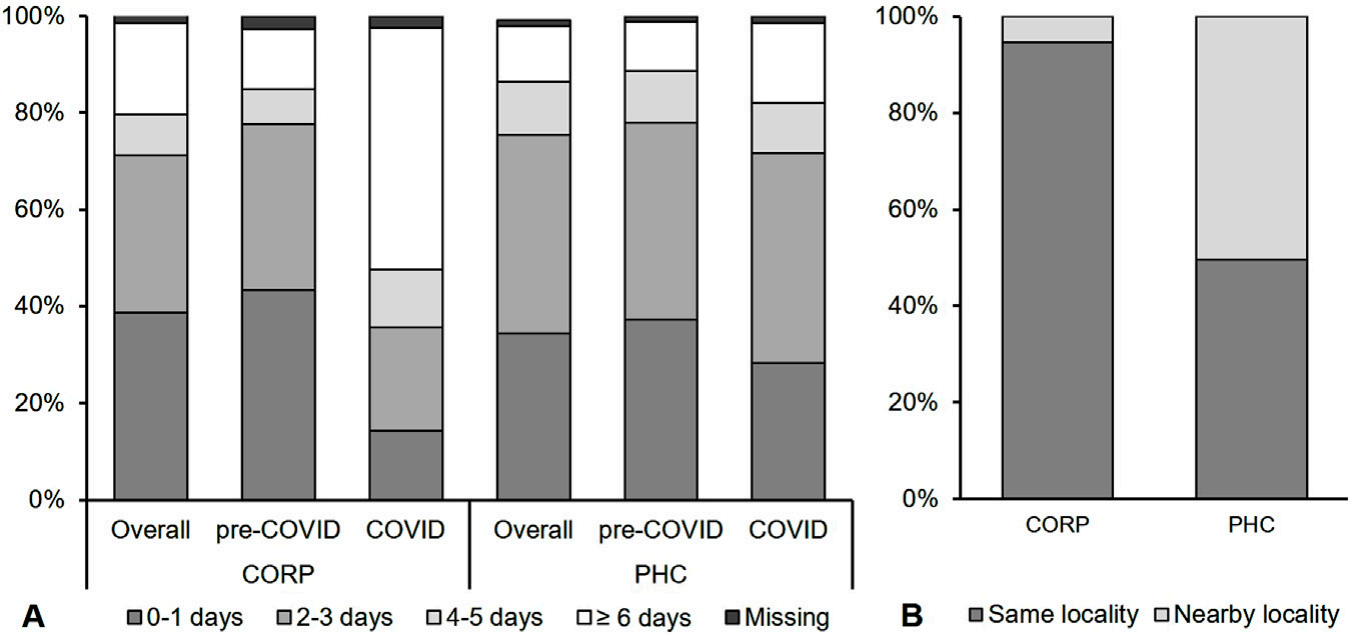
Treatment seeking at community-based providers: **(A)** Time between illness onset and visiting the respective provider in days by enrolment period; **(B)** Location of the enrolling provider in relation to the child’s residence (Adamawa State, Nigeria. 2018–2020).

Further treatment seeking details were analysed for 264 children who attended a CORP and 242 who attended a PHC. There was no substantial difference in the number of days between illness onset and visiting a CORP or a PHC (Figure 2A). Among children attending a CORP, 38% attended the same day or the day after illness onset and 32% 2–3 days after becoming ill. Among PHC enrolments, slightly more children attended the PHC 2–3 days after symptom onset (41%) compared to the same day or day after (35%). Home treatment was associated with a longer treatment seeking delay. Children for whom prompt care was sought had the lowest rates of home treatment administration (CORP: 18% and PHC: 32%) compared to those with delays greater than 1 day (CORP: 44% and PHC: 52%; p < 0.01). Before visiting the enrolling CORP or PHC, 14% of children attending a PHC and 8% of those attending a CORP had been to another provider (AOR: 2.2, 95% CI: 1.1–4.4). In most cases, the previous provider was a chemist/pharmacy/drug shop for both CORP (47%, 9/19) and PHC (71%, 24/34) enrolments (Fisher’s test p = 0.85). The COVID-19 pandemic was found to decrease the proportion seeking timely treatment (same or following day) from CORPs (AOR: 5.1, 95% CI: 1.8–14.3) but not from PHCs (AOR: 1.5, 95% CI: 0.7–3.1).

PHCs, offering a wider range of services and being much fewer than CORPs, were significantly more likely to be located outside the child’s locality than CORPs (50% vs. 6%; AOR: 18.0, 95% CI: 6.8–47.2) (Figure 2B).

**FIGURE 3:**
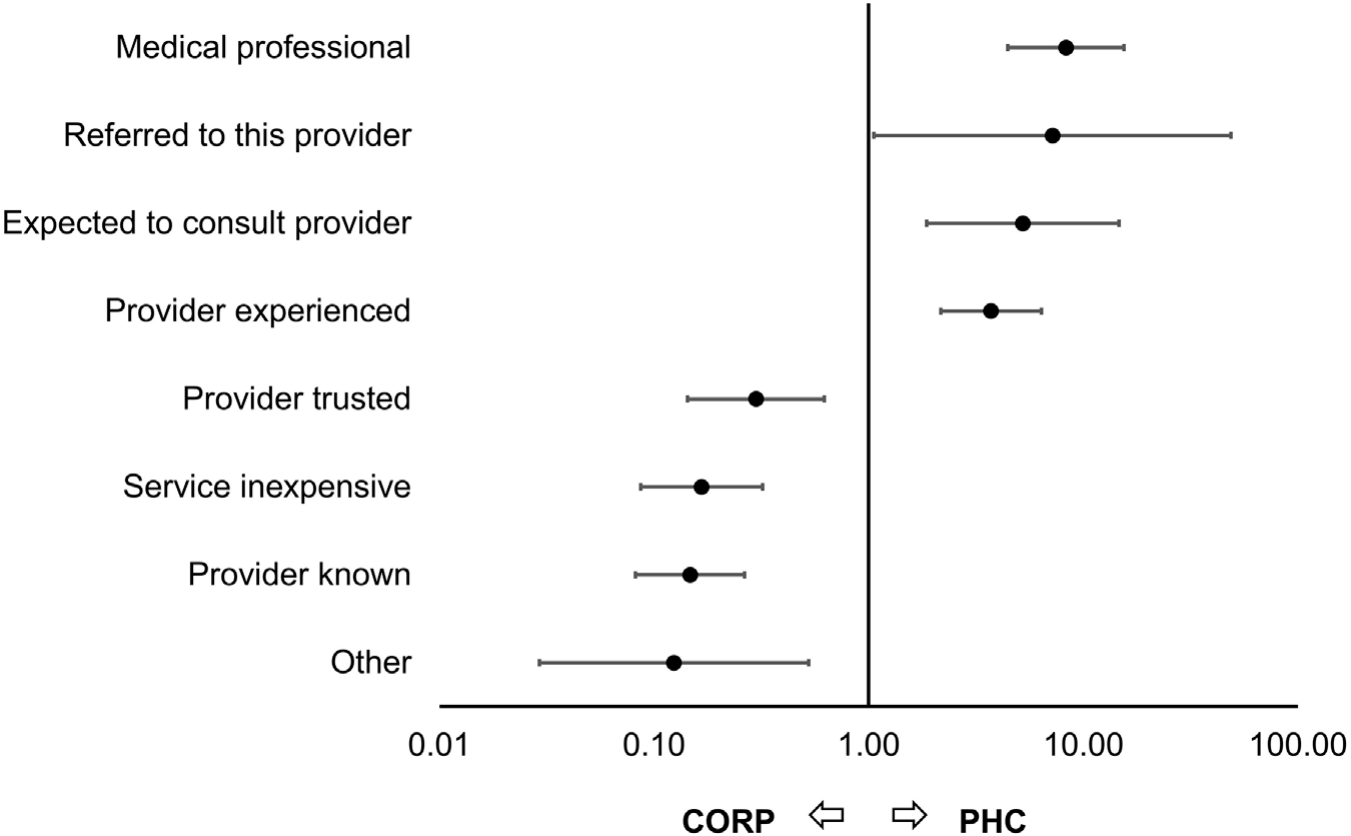
Odds ratios for reasons caregivers took their child with severe febrile illness to a Primary Health Centre (PHC) compared to a Community Oriented Resource Person (CORP) (Adamawa State, Nigeria. 2018–2020). Logistic regression adjusting for the COVID-19 pandemic, with standard errors clustered at the level of the healthcare provider.

A total of 456 caregivers provided reasons for taking their child to a CORP or a PHC (Figure 3). The most common reasons for visiting a CORP were knowing (76%) and trusting (26%) the health worker and low cost (22%). In contrast, reasons for attending a PHC included the experience (49%) and medical professionalism (34%) of PHC health workers, as well as knowing the facility (32%). Knowing or trusting the provider and the service being inexpensive were significantly more common among caregivers who brought their child to a CORP, whereas experience and professionalism of the provider and an expectation or custom to consult this provider were more frequent reasons for attending a PHC.

## Discussion

Severely sick children need prompt diagnosis and effective clinical management to prevent long-lasting sequelae or death [6]. Diagnostic algorithms such as iCCM and IMCI should allow rapid triaging of severely sick patients close to a patient’s home, and ensure administration of pre-referral treatment and transfer to a higher-level health facility. In Nigeria’s Adamawa State, we found that children with suspected severe malaria who attended a PHC were three times more likely to die than those who visited a CORP (CFR 18.5% vs. 5.7%, [9]), a finding that could not be explained by differences in referral delay or post-referral antimalarial treatment [9]. We therefore investigated differences in background characteristics and treatment history of patients who attended a CORP or a PHC, respectively, to better understand the observed difference in CFR.

There were no significant differences in demographic characteristics of patients attending CORPs or PHCs. However, those attending PHCs lived in more urban areas, which may reflect socio-demographic differences not directly visible in our data. Children at PHCs also appeared to be more severely ill as reflected in the presence of CNS danger signs (90% vs. 74% in those attending CORPs). As the observational study design did not allow for an expert clinical examination, the presence of CNS symptoms was used as a proxy for severe disease, as previously done in similar situations [23–26]. Severity was also reflected in more children attending a PHC having ≥4 clinical danger signs compared to children taken to a CORP. While this analysis considered danger signs on which CORPs were specifically trained, the more thorough clinical training of health workers at PHCs may have biased the recognition of danger signs. Yet, while danger signs indicated that more severely ill children were rather brought to a PHC, this was not reflected in higher caregiver-perceived illness severity, though this study did not explore multidimensional aspects of perceived severity (e.g. emotions, knowledge about severe malaria, economic consequences in seeking or not seeking treatment) [13].

It is plausible that more children with specific signs of severity are rather brought to a PHC than to a CORP as caregivers assume higher professionalism and experience of providers at PHCs. Greater illness severity increased the likelihood of care seeking at health centres compared to community-health workers and self-care in Papua New Guinea [17]. Warsame et al. (2016) previously found that children attending community health workers, in settings comparable to ours, had higher rates of CNS symptoms compared to those attending other health providers such as trained mothers or traditional healers, suggesting higher perceived professional capacity [26]. In Burkina Faso, cases of severe disease often bypass community health workers in favour of a professional health worker [27]. CNS symptoms and difficulties in breathing as signs of severity in febrile children have also been associated with hospital attendance in rural Tanzania [28].

Initial home treatment (usually an antipyretic, rarely an antimalarial) and first attending another provider (mostly a drug shop/chemist/pharmacy) were more common among children brought to a PHC compared to those brought to a CORP. This pattern may reflect a staged escalation in the level of care if symptoms do not improve. As observed elsewhere [29], and also in our study, home treatment and attending medicine retailers did not facilitate access to appropriate medicines but more likely delayed the administration of appropriate treatment by a qualified healthcare provider. As a result, an uncomplicated malaria episode may have progressed to severe malaria, or urgent treatment of an already severe illness episode may have been delayed In Nigeria, lower quality providers are more often sought for children of lower socioeconomic status [30–32] and most drug sellers are not integrated in the formal health system and the continuum of care for severe malaria (for instance, they do not administer pre-referral RAS).

Two-thirds of the children in our study were not brought to the PHC or CORP on the day of onset of symptoms or the day after. Whether delay in attending a healthcare provider has contributed to the overall high CFR in Adamawa is unclear since we lack information on how quickly a particular episode became severe. Yet, it is known that treatment seeking delays increase the risk of progressing from uncomplicated to severe malaria [33] and mortality is highest within 24 h of onset of severe disease [12]. Distance and transport time may negatively affect the condition of a severely sick child especially if transport is strenuous. In Zambia, greater distance from the hospital increased the odds of death in children with severe malaria [34].

We did not find a significant difference in treatment seeking delay between children attending a CORP or PHC, though the retrospective assessment with a 28 days recall period may have limited how precisely time periods were recalled. Thus, some nuances in treatment delay may have remained undetected. During COVID-19 pandemic movement restrictions, delay in seeking treatment increased substantially among those going to a CORP but only marginally among those attending a PHC. CORPs experienced stockouts of commodities and delay in replenishment during the COVID-19 lockdown (own observations), which could have discouraged attendance. Additionally, over the course of the study, behavioural change communication campaigns were conducted alongside RAS roll-out to sensitize communities [35] and may have contributed to caregivers’ awareness about severe malaria and perception about health providers’ capabilities in treating their child’s illness.

Other factors may have influenced the choice of community-based providers without necessarily affecting the CFR. This includes the distance between a patient’s home and the nearest CORPs, PHCs, and referral facilities, as well as the distance of these health providers to one another. Druetz et al. showed that in rural Burkina Faso, consulting a community health worker for malaria was associated with increased distance to the nearest health facility [36]. Provider operating hours also affect health-seeking behaviours. We found that on weekends, patients rather attended CORPs than PHCs that did not provide 24-hour service. A different finding was published by Koce et al. who found that in Nigeria, the limited staff and operating hours of community-based providers (compared to referral facilities) resulted in patients directly seeking care from hospitals, which usually operate 24 h and have medical doctors [37].

Taken together, our findings suggest that, compared to those attending a CORP, children who attended a PHC may have been more severely ill, potentially aggravated by inadequate home management, delay in attending the health facility, and possibly a longer and more strenuous travel. As recognition of malaria and severe disease is often poor [38], caregivers may have underestimated the severity of the child’s condition even in the presence of danger signs. Combined with a low capacity of PHCs to manage severe illness, these factors may have contributed to a higher CFR among children attending a PHC; however, they are unlikely to be the only explanation. Other factors affecting the health outcome of children in this study were investigated in separate analyses. For example, the likelihood of death was higher among children who were treated with pre-referral RAS (AOR: 2.42, 95% CI: 1.25–4.70), whereas a higher proportion of children attending PHCs received RAS compared to those attending CORPs [9]. This finding may be related to incomplete referral and the quality of post-referral care rather than to the administration of RAS itself. For example, only 48% of children completed referral to a Cottage Hospital after administration of RAS by a CORP or PHC, though children attending a PHC were much more likely to complete referral [18]. While hospitalized, injectable antimalarial treatment was common but <4% of children were subsequently treated with the required oral ACT [19]. Several suspected viral outbreaks occurred in Adamawa State during the study period, including Lassa Fever and Yellow Fever [39, 40]. The symptoms of these diseases are often unspecific and overlap with severe malaria, while health providers at all levels in remote locations often lack diagnostic capacity for these infections [41]. Over-diagnosis of severe malaria in children with severe febrile illness is also a well-known issue [42, 43]. While all children included in this analysis had a positive malaria RDT, severe illness and eventually death may have resulted from a missed or inappropriately managed co-infection. Further support for this is that the time between visiting the community-based provider and death was 6.3 days in Nigeria [9], however, the risk of death due to severe malaria is highest early on. Our findings hence indicate that children in Adamawa State with a severe febrile illness indicative of severe malaria face a high risk of dying even if they attend formal primary healthcare services. Several factors along the continuum of care are likely to contribute to the high proportion of deaths in children attending a PHC, including the inadequate quality of treatment received at higher level referral facilities.

In the broader context, these findings underscore the necessity to not only improve access to primary healthcare services and prompt treatment initiation, but to implement health systems strengthening measures to improve referral systems and quality of care at all levels [44]. These considerations, based on findings in this publication and others emerging from the CARAMAL project, were presented at a technical consultation convened by the WHO with the aim to develop operational guidance for an effective implementation of pre-referral RAS in community-based health systems [45].

This study contributes to literature on care seeking for severely ill children, a population which is comparably small and for which published evidence is scarce [13]. While findings from this study may not necessarily be directly applicable to other settings, they point to community level service delivery issues that warrant careful attention when promoting the management of severe childhood illness in African settings.

In conclusion, this research provides context on treatment seeking for children with suspected severe malaria in a northern Nigerian setting, where children visiting a PHC were significantly more likely to die than those attending a CORP. In the context of weak healthcare systems, delay in treatment seeking, higher illness severity in children visiting PHCs, and the inability of PHCs to provide appropriate care, are all likely important drivers of high case fatality. Promoting prompt treatment seeking and strengthening PHCs, referral, and post-referral care are all critical to prevent deaths in children attending community healthcare services.

## Data Availability

The datasets generated and/or analysed during the current study are available from the Zenodo repository (https://doi.org/10.5281/zenodo.5733005) upon reasonable request to the corresponding author.

## Abbreviations

ACT, Artemisinin-based Combination Therapy; AOR, Adjusted Odds Ratio; CARAMAL, Community Access to Rectal Artesunate for Malaria; CFR, Case Fatality Rate; CNS, Central Nervous System; CORP, Community Oriented Resource Person; iCCM, integrated Community Case Management; IMCI, Integrated Management of Childhood Illnesses; LGA, Local Government Areas; mRDT, malaria Rapid Diagnostic Test; PHC, Primary Health Center; RAS, Rectal Artesunate; WHO, World Health Organization.

## References

[1] World Health Organization. World Malaria Report 2024: Addressing Inequity in the Global Malaria Response. Geneva: World Health Organization (2024).

[2] Abubakar I Dalglish SL Angell B Sanuade O Abimbola S Adamu AL The Lancet Nigeria Commission: Investing in Health and the Future of the Nation. Lancet (2022) 399(10330):1155–200. 10.1016/S0140-6736(21)02488-0 35303470 PMC8943278

[3] Kendjo E Agbenyega T Bojang K Newton CR Bouyou-Akotet M Pedross F Mortality Patterns and Site Heterogeneity of Severe Malaria in African Children. PLoS One (2013) 8(3):e58686. 10.1371/journal.pone.0058686 23505549 PMC3591404

[4] Bassat Q Guinovart C Sigaúque B Aide P Sacarlal J Nhampossa T Malaria in Rural Mozambique. Part II: Children Admitted to Hospital. Malar J (2008) 7:37. 10.1186/1475-2875-7-37 18302771 PMC2275288

[5] Orimadegun AE Fawole O Okereke JO Akinbami FO Sodeinde O. Increasing Burden of Childhood Severe Malaria in a Nigerian Tertiary Hospital: Implication for Control. J Trop Pediatr (2007) 53(3):185–9. 10.1093/tropej/fmm002 17287244

[6] World Health Organization. Guidelines for the Treatment of Malaria. 3rd ed. (2015). 26020088

[7] Health Economic Finance Development Consortium. Integrated Community Case Management (iCCM) in Sub-Saharan Africa: Successes and Challenges with Access, Speed and Quality. Nairobi: Health Economic Finance Development Consortium (2018).

[8] Lengeler C Burri C Awor P Athieno P Kimera J Tumukunde G Community Access to Rectal Artesunate for Malaria (CARAMAL): A Large-Scale Observational Implementation Study in the Democratic Republic of the Congo, Nigeria and Uganda. PLOS Glob Public Health (2022) 2(9):e0000464. 10.1371/journal.pgph.0000464 36962706 PMC10022208

[9] Hetzel MW Okitawutshu J Tshefu A Omoluabi E Awor P Signorell A Effectiveness of Rectal Artesunate as Pre-Referral Treatment for Severe Malaria in Children Under 5 Years of Age: A Multi-Country Observational Study. BMC Med (2022) 20(1):343–12. 10.1186/s12916-022-02541-8 36217159 PMC9550309

[10] Gomes MF Faiz MA Gyapong JO Warsame M Agbenyega T Babiker A Pre-Referral Rectal Artesunate to Prevent Death and Disability in Severe Malaria: A Placebo-Controlled Trial. Lancet (2009) 373(9663):557–66. 10.1016/S0140-6736(08)61734-1 19059639 PMC2646124

[11] Poespoprodjo JR Douglas NM Ansong D Kho S Anstey NM. Malaria. Lancet. (2023) 402(10419):2328–45. 10.1016/S0140-6736(23)01249-7 37924827

[12] World Health Organization. Severe Malaria. Trop Med Int Health (2014) 19(Suppl. 1):7–131. 10.1111/tmi.12313_2 25214480

[13] Brunner NC Awor P Hetzel MW. Definitions of Severity in Treatment Seeking Studies of Febrile Illness in Children in Low and Middle Income Countries: A Scoping Review. Int J Public Health (2021) 66(74):634000. 10.3389/ijph.2021.634000 34526874 PMC8435535

[14] Tinuade O Iyabo RA Durotoye O. Health-Care-Seeking Behaviour for Childhood Illnesses in a Resource-Poor Setting. J Paediatr Child Health (2010) 46(5):238–42. 10.1111/j.1440-1754.2009.01677.x 20337870

[15] Adah OS Ngo-Ndomb T Envuladu EA Audu S Banwat ME Yusuff OT Home Treatment of Malaria, Amongst Under Fives Presenting with Fever in PHC Facilities in Jos North LGA of Plateau State. Niger J Med (2009) 18(1):88–93. 19485157

[16] Ellis AA Traore S Doumbia S Dalglish SL Winch PJ. Treatment Actions and Treatment Failure: Case Studies in the Response to Severe Childhood Febrile Illness in Mali. BMC Public Health (2012) 12:946. 10.1186/1471-2458-12-946 23127128 PMC3497867

[17] Tsukahara T Ogura S Sugahara T Sekihara M Furusawa T Kondo N The Choice of Healthcare Providers for Febrile Children After Introducing Non-Professional Health Workers in a Malaria Endemic Area in Papua New Guinea. Front Public Health (2015) 3:275. 10.3389/fpubh.2015.00275 26734599 PMC4689805

[18] Brunner NC Omoluabi E Awor P Okitawutshu J Kitoto AT Signorell A Prereferral Rectal Artesunate and Referral Completion Among Children with Suspected Severe Malaria in the Democratic Republic of the Congo, Nigeria and Uganda. BMJ Glob Health (2022) 7(5):e008346. 10.1136/bmjgh-2021-008346 35580913 PMC9114942

[19] Signorell A Awor P Okitawutshu J Tshefu A Omoluabi E Hetzel MW Health Worker Compliance with Severe Malaria Treatment Guidelines in the Context of Implementing Pre-Referral Rectal Artesunate in the Democratic Republic of the Congo, Nigeria, and Uganda: An Operational Study. PLoS Med (2023) 20(2):e1004189. 10.1371/journal.pmed.1004189 36809247 PMC9990943

[20] WorldPop. World Population Demographics: Subnational Age/Sex Structures - 2000-2020 2018. Available online at: https://www.portal.worldpop.org/demographics/ (Accessed June 20, 2024).

[21] Weber EM Seaman VY Stewart RN Bird TJ Tatem AJ McKee JJ Census-Independent Population Mapping in Northern Nigeria. Remote Sens Environ (2018) 204:786–98. 10.1016/j.rse.2017.09.024 29302127 PMC5738969

[22] Abadie A Athey S Imbens GW Wooldridge J. When Should You Adjust Standard Errors for Clustering? National Bureau of Economic Research Working Paper Series No. 24003 (2017). 10.3386/w24003

[23] Simba DO Warsame M Kimbute O Kakoko D Petzold M Tomson G Factors Influencing Adherence to Referral Advice Following Pre-Referral Treatment with Artesunate Suppositories in Children in Rural Tanzania. Trop Med Int Health (2009) 14(7):775–83. 10.1111/j.1365-3156.2009.02299.x 19497077

[24] Siribie M Ajayi IO Nsungwa-Sabiiti J Sanou AK Jegede AS Afonne C Compliance with Referral Advice After Treatment with Prereferral Rectal Artesunate: A Study in 3 Sub-Saharan African Countries. Clin Infect Dis (2016) 63(Suppl. 5):S283-S289–S9. 10.1093/cid/ciw627 27941106 PMC5146699

[25] Ajayi IO Nsungwa-Sabiiti J Siribié M Falade CO Sermé L Balyeku A Feasibility of Malaria Diagnosis and Management in Burkina Faso, Nigeria, and Uganda: A Community-Based Observational Study. Clin Infect Dis (2016) 63(Suppl. 5):S245–S255. 10.1093/cid/ciw622 27941101 PMC5146694

[26] Warsame M Gyapong M Mpeka B Rodrigues A Singlovic J Babiker A Pre-Referral Rectal Artesunate Treatment by Community-Based Treatment Providers in Ghana, Guinea-Bissau, Tanzania, and Uganda (Study 18): A Cluster-Randomized Trial. Clin Infect Dis (2016) 63(Suppl. 5):S312–S321. 10.1093/cid/ciw631 27941110 PMC5146703

[27] Sauerborn R Nougtara A Diesfeld HJ. Low Utilization of Community Health Workers: Results from a Household Interview Survey in Burkina Faso. Soc Sci Med (1989) 29(10):1163–74. 10.1016/0277-9536(89)90359-6 2588044

[28] Castellani J Mihaylova B Evers SM Paulus AT Mrango ZE Kimbute O Out-Of-Pocket Costs and Other Determinants of Access to Healthcare for Children with Febrile Illnesses: A Case-Control Study in Rural Tanzania. PLoS One (2015) 10(4):e0122386. 10.1371/journal.pone.0122386 25861012 PMC4393118

[29] O'Connell KA Gatakaa H Poyer S Njogu J Evance I Munroe E Got Acts? Availability, Price, Market Share and Provider Knowledge of Anti-Malarial Medicines in Public and Private Sector Outlets in Six Malaria-Endemic Countries. Malar J (2011) 10:326. 10.1186/1475-2875-10-326 22039838 PMC3227612

[30] Onwujekwe O Hanson K Uzochukwu B. Do Poor People Use Poor Quality Providers? Evidence from the Treatment of Presumptive Malaria in Nigeria. Trop Med Int Health (2011) 16(9):1087–98. 10.1111/j.1365-3156.2011.02821.x 21702870

[31] Onwujekwe O Hanson K Uzochukwu B Ezeoke O Eze S Dike N. Geographic Inequities in Provision and Utilization of Malaria Treatment Services in Southeast Nigeria: Diagnosis, Providers and Drugs. Health Policy (2010) 94(2):144–9. 10.1016/j.healthpol.2009.09.010 19836852

[32] Isiguzo C Anyanti J Ujuju C Nwokolo E De La Cruz A Schatzkin E Presumptive Treatment of Malaria from Formal and Informal Drug Vendors in Nigeria. PLoS One (2014) 9(10):e110361. 10.1371/journal.pone.0110361 25333909 PMC4204870

[33] Mousa A Al-Taiar A Anstey NM Badaut C Barber BE Bassat Q The Impact of Delayed Treatment of Uncomplicated P. falciparum Malaria on Progression to Severe Malaria: A Systematic Review and a Pooled Multicentre Individual-Patient Meta-Analysis. PLoS Med (2020) 17(10):e1003359. 10.1371/journal.pmed.1003359 33075101 PMC7571702

[34] Ippolito MM Kamavu LK Kabuya JB Tente C Chileshe E Wapachole M Risk Factors for Mortality in Children Hospitalized with Severe Malaria in Northern Zambia: A Retrospective Case-Control Study. Am J Trop Med Hyg (2018) 98(6):1699–704. 10.4269/ajtmh.17-1017 29692306 PMC6086172

[35] Lengeler C Burri C Awor P Athieno P Kimera J Tumukunde G Community Access to Rectal Artesunate for Malaria (CARAMAL): A Large-Scale Observational Implementation Study in the Democratic Republic of the Congo, Nigeria and Uganda. (2021). 10.1371/journal.pgph.0000464 PMC10022208 36962706

[36] Druetz T Ridde V Kouanda S Ly A Diabaté S Haddad S. Utilization of Community Health Workers for Malaria Treatment: Results from a Three-Year Panel Study in the Districts of Kaya and Zorgho, Burkina Faso. Malar J (2015) 14:71. 10.1186/s12936-015-0591-9 25889306 PMC4329655

[37] Koce FG Randhawa G Ochieng B. A Qualitative Study of Health Care Providers’ Perceptions and Experiences of Patients Bypassing Primary Healthcare Facilities: A Focus from Nigeria. J Glob Health Rep (2020) 4. 10.29392/001c.14138

[38] Elimian KO Myles PR Phalkey R Sadoh A Pritchard C. Comparing the Accuracy of Lay Diagnosis of Childhood Malaria and Pneumonia with that of the Revised IMCI Guidelines in Nigeria. J Public Health (Oxf) (2020) 43:772–9. 10.1093/pubmed/fdaa103 32671386

[39] World Health Organization. Yellow Fever – Nigeria. (2021).

[40] Agbonlahor DE Akpede GO Happi CT Tomori O. 52 Years of Lassa Fever Outbreaks in Nigeria, 1969-2020: An Epidemiologic Analysis of the Temporal and Spatial Trends. Am J Trop Med Hyg (2021) 105:974–85. 10.4269/ajtmh.20-1160 34460421 PMC8592130

[41] Roddy P Dalrymple U Jensen TO Dittrich S Rao VB Pfeffer DA Quantifying the Incidence of Severe-Febrile-Illness Hospital Admissions in Sub-Saharan Africa. PLoS One (2019) 14(7):e0220371. 10.1371/journal.pone.0220371 31344116 PMC6657909

[42] Gwer S Newton CR Berkley JA. Over-Diagnosis and Co-Morbidity of Severe Malaria in African Children: A Guide for Clinicians. Am J Trop Med Hyg (2007) 77(6 Suppl. l):6–13. 10.4269/ajtmh.2007.77.6 18165469 PMC2669774

[43] Stoler J Awandare GA. Febrile Illness Diagnostics and the malaria-industrial Complex: A Socio-Environmental Perspective. BMC Infect Dis (2016) 16(1):683. 10.1186/s12879-016-2025-x 27855644 PMC5114833

[44] Hetzel MW Awor P Tshefu A Omoluabi E Burri C Signorell A Pre-Referral Rectal Artesunate: No Cure for Unhealthy Systems. Lancet Infect Dis (2023) 23(6):e213–e217. 10.1016/S1473-3099(22)00762-9 36549311

[45] World Health Organization. Technical Consultation to Review the Effectiveness of Rectal Artesunate Used as Pre-referral Treatment of Severe Malaria in Children: Meeting Report, 18–19 October 2022. Geneva: World Health Organization (2023).

