# Supplementary material for: Treatment-Seeking for Children with Suspected Severe Malaria Attending Community Health Workers and Primary Health Centres in Adamawa State, Nigeria
Source: Int J Public Health. 2025 Sep 26;70:1607697. doi: 10.3389/ijph.2025.1607697 (PMC12510906; doi:10.3389/ijph.2025.1607697)
Supplement: Supplementary file 1 [file Supplementaryfile1.docx]

**Supplemental materials**

**Figure S1. Inclusion flow chart**

**Table S1. Study population characteristics by enrolling provider with unadjusted estimates**

|  | **CORP** | |  | **PHC** | |  |  |  |  |
| --- | --- | --- | --- | --- | --- | --- | --- | --- | --- |
|  | **N** | **%** |  | **N** | **%** |  | **OR*** | **95% CI*** | **p-value*** |
| **Total** | **314** |  |  | **275** |  |  |  |  |  |
| **Child sex** |  |  |  |  |  |  |  |  |  |
| Male | 188 | 60 |  | 164 | 60 |  | Ref. |  |  |
| Female | 126 | 40 |  | 111 | 40 |  | 1.0 | (0.7 - 1.4) | 0.95 |
| **Child age (years)** |  |  |  |  |  |  |  |  |  |
| 0 | 40 | 13 |  | 27 | 10 |  | Ref. |  | 0.76 |
| 1 | 86 | 27 |  | 74 | 27 |  | 1.3 | (0.6 - 2.5) |  |
| 2 | 82 | 26 |  | 82 | 30 |  | 1.5 | (0.8 - 2.8) |  |
| 3 | 68 | 22 |  | 55 | 20 |  | 1.2 | (0.6 - 2.3) |  |
| 4 | 38 | 12 |  | 37 | 13 |  | 1.4 | (0.6 - 3.2) |  |
| **Caregiver sex** |  |  |  |  |  |  |  |  |  |
| Male | 102 | 32 |  | 95 | 35 |  | Ref. |  |  |
| Female | 212 | 68 |  | 180 | 65 |  | 0.9 | (0.6 - 1.3) | 0.64 |
| **Caregiver age** |  |  |  |  |  |  |  |  |  |
| < 25 | 42 | 13 |  | 46 | 17 |  | Ref. |  | 0.47 |
| 25 - 34 | 167 | 53 |  | 128 | 47 |  | 0.7 | (0.4 - 1.2) |  |
| 35 - 45 | 75 | 24 |  | 72 | 26 |  | 0.9 | (0.5 - 1.6) |  |
| ≥ 45 | 30 | 10 |  | 28 | 10 |  | 0.9 | (0.4 - 1.6) |  |
| Missing | 0 | 0 |  | 1 | 0 |  | - |  |  |
| **Caregiver education**** |  |  |  |  |  |  |  |  |  |
| Never attended school | 54 | 17 |  | 65 | 24 |  | Ref. |  | 0.63 |
| Primary or lower | 15 | 5 |  | 26 | 9 |  | 1.4 | (0.6 - 3.3) |  |
| Secondary or higher | 28 | 9 |  | 44 | 16 |  | 1.3 | (0.6 - 2.6) |  |
| Quranic | 22 | 7 |  | 39 | 14 |  | 1.5 | (0.7 - 3.1) |  |
| Missing | 195 | 62 |  | 101 | 37 |  | - |  |  |
| **LGA** |  |  |  |  |  |  |  |  |  |
| Fufore | 143 | 46 |  | 94 | 34 |  | Ref. |  | 0.12 |
| Mayo-Belwa | 95 | 30 |  | 154 | 56 |  | 2.5 | (0.6 - 11.0) |  |
| Song | 76 | 24 |  | 27 | 10 |  | 0.5 | (0.1 - 2.0) |  |
| **Residence** |  |  |  |  |  |  |  |  |  |
| Rural | 298 | 95 |  | 227 | 83 |  | Ref. |  |  |
| Urban | 14 | 4 |  | 43 | 16 |  | 4.0 | (1.2 - 13.5) | 0.02 |
| Missing | 2 | 1 |  | 5 | 2 |  | - |  |  |
| **Day of enrolment** |  |  |  |  |  |  |  |  |  |
| Workday | 241 | 77 |  | 239 | 87 |  | Ref. |  |  |
| Weekend | 73 | 23 |  | 36 | 13 |  | 0.5 | (0.3 - 0.9) | 0.02 |
| **Season** |  |  |  |  |  |  |  |  |  |
| Dry season | 83 | 26 |  | 66 | 24 |  | Ref. |  |  |
| Rainy season | 231 | 74 |  | 209 | 76 |  | 1.1 | (0.7 - 1.9) | 0.63 |
| **RAS implementation phase** |  |  |  |  |  |  |  |  |  |
| Pre-RAS | 156 | 50 |  | 61 | 22 |  | Ref. |  |  |
| Post-RAS | 158 | 50 |  | 214 | 78 |  | 3.5 | (1.8 - 6.7) | <0.01 |
| **COVID-19 pandemic** |  |  |  |  |  |  |  |  |  |
| Pre-COVID-19 | 268 | 85 |  | 207 | 75 |  | Ref. |  |  |
| COVID-19 period | 46 | 15 |  | 68 | 25 |  | 1.9 | (1.0 - 3.6) | 0.04 |

*Unadjusted logistic regression with standard errors clustered at the level of the health care provider. Likelihood ratio test used to calculate p-values for categorical variables. **Data not collect in collected during the complete study period.

**Table S2. Signs and symptoms of disease and caregiver’s perceived severity of the illness with unadjusted estimates**

|  | **CORP** | |  | **PHC** | |  |  |  |  |
| --- | --- | --- | --- | --- | --- | --- | --- | --- | --- |
|  | **N** | **%** |  | **N** | **%** |  | **OR*** | **95% CI*** | **p-value*** |
| **Total** | **314** |  |  | **275** |  |  |  |  |  |
| **RAS danger sign** |  |  |  |  |  |  |  |  |  |
| Convulsions | 170 | 54 |  | 217 | 79 |  | 3.2 | (2.0 - 5.0) | <0.01 |
| Not able to drink or feed anything | 200 | 64 |  | 159 | 58 |  | 0.8 | (0.5 - 1.1) | 0.18 |
| Vomits everything | 227 | 72 |  | 160 | 58 |  | 0.5 | (0.4 - 0.8) | <0.01 |
| Unusually sleepy or unconscious | 175 | 56 |  | 193 | 70 |  | 1.9 | (1.3 - 2.7) | <0.01 |
|  | **117** |  |  | **172** |  |  |  |  |  |
| Yellowness of the eyes ** | 8 | 7 |  | 4 | 2 |  | 0.3 | (0.1 - 1.1) | 0.06 |
| **Other danger signs** | **314** |  |  | **275** |  |  |  |  |  |
| Blood in stool | 57 | 18 |  | 20 | 7 |  | 0.4 | (0.2 - 0.6) | <0.01 |
| Swelling of both feet | 5 | 2 |  | 11 | 4 |  | 2.6 | (0.8 - 8.6) | 0.12 |
| Unable to sit or stand | 140 | 45 |  | 147 | 53 |  | 1.4 | (1.0 - 2.0) | 0.04 |
|  | **117** |  |  | **172** |  |  |  |  |  |
| Cough for 14 days or more ** | 3 | 3 |  | 3 | 2 |  | 0.7 | (0.1 - 3.2) | 0.62 |
| Diarrhoea for 14 days or more ** | 4 | 3 |  | 1 | 1 |  | 0.2 | (0.0 - 1.3) | 0.09 |
| Fever lasting 7 days or more ** | 57 | 49 |  | 53 | 31 |  | 0.6 | (0.4 - 1.0) | 0.05 |
| Whiteness of the palms and sole ** | 3 | 3 |  | 14 | 8 |  | 3.4 | (0.9 - 12.6) | 0.07 |
| Coke coloured urine** | 26 | 22 |  | 48 | 28 |  | 1.4 | (0.7 - 2.7) | 0.38 |
| Number of danger signs *** | **314** |  |  | **275** |  |  |  |  |  |
| 0 - 1 | 52 | 17 |  | 32 | 12 |  | Ref. |  | 0.06 |
| 2 - 3 | 138 | 44 |  | 106 | 39 |  | 1.2 | (0.7 - 2.1) |  |
| ≥ 4 | 124 | 39 |  | 137 | 50 |  | 1.8 | (1.1 - 3.0) |  |
| **CNS  danger sign (unusually sleepy or unconscious or convulsions)** | 231 | 74 |  | 248 | 90 |  | 3.3 | (1.9 - 5.7) | <0.01 |
| **Perceived severity of the illness** |  |  |  |  |  |  |  |  |  |
| Not fatal | 236 | 75 |  | 193 | 70 |  | Ref. |  |  |
| Fatal | 76 | 24 |  | 79 | 29 |  | 1.3 | (0.8 - 2.0) | 0.28 |
| Don’t know/Missing | 2 | 1 |  | 3 | 1 |  | - |  |  |

*Unadjusted logistic regression with standard errors clustered at the level of the health care provider. Likelihood ratio test used to calculate p-values for categorical variables. **3 children were not said to have any danger signs by caregivers at follow-up but health providers noted danger signs at enrolment. ***Only includes danger signs collected across the whole study period.

**Table S3. Actions taken at home prior to consulting health providers**

|  | **CORP** | |  | **PHC** | |  |  |  |  |
| --- | --- | --- | --- | --- | --- | --- | --- | --- | --- |
|  | **N** | **%** |  | **N** | **%** |  | **OR*** | **95% CI*** | **p-value*** |
| **Total** | **314** |  |  | **275** |  |  |  |  |  |
| **Any home treatment** |  |  |  |  |  |  |  |  |  |
| No | 209 | 67 |  | 157 | 57 |  | Ref. |  |  |
| Yes | 103 | 33 |  | 116 | 42 |  | 1.5 | (1.0 - 2.2) | 0.03 |
| Missing | 2 | 1 |  | 2 | 1 |  | - |  |  |
| **Actions taken** | **103** |  |  | **116** |  |  |  |  |  |
| Traditional medicines/herbs | 22 | 21 |  | 26 | 22 |  | 1.1 | (0.5 - 2.2) | 0.87 |
| Tepid sponging | 7 | 7 |  | 17 | 15 |  | 2.4 | (0.9 - 6.4) | 0.09 |
| Given medicine | 79 | 77 |  | 85 | 73 |  | 0.8 | (0.5 - 1.5) | 0.55 |
| **Caregiver remembers what medicine the child received** | **79** |  |  | **85** |  |  |  |  |  |
| No | 12 | 15 |  | 9 | 11 |  | Ref. |  |  |
| Yes | 67 | 85 |  | 76 | 89 |  | 1.5 | (0.6 - 4.0) | 0.40 |
| **Medicines given** | **67** |  |  | **76** |  |  |  |  |  |
| Artemether-lumefantrine | 10 | 15 |  | 11 | 14 |  | 1.0 | (0.3 - 3.1) | 0.95 |
| Artesunate-amodiaquine | 0 | 0 |  | 0 | 0 |  | - |  | - |
| Oral rehydration solution (ORS) | 1 | 1 |  | 1 | 1 |  | 0.9 | (0.1 - 12.9) | 0.93 |
| Paracetamol | 57 | 85 |  | 70 | 92 |  | 2.0 | (0.6 - 6.9) | 0.25 |
| Other | 6 | 9 |  | 5 | 7 |  | 0.7 | (0.2 - 2.6) | 0.61 |

*Unadjusted logistic regression with standard errors clustered at the level of the health care provider. Likelihood ratio test used to calculate p-values for categorical variables.
